# Clinical characteristics and prognostic factors of COVID-19 infection among cancer patients during the December 2022 – February 2023 Omicron variant outbreak

**DOI:** 10.3389/fmed.2024.1401439

**Published:** 2024-05-30

**Authors:** Li-Li Liu, Yu-Wei Liao, Xiao-Hua Yu, Ling Rong, Bi-Gui Chen, Gang Chen, Guang-Kuan Zeng, Li-Ye Yang

**Affiliations:** ^1^Precision Medical Laboratory Center, People’s Hospital of Yangjiang Affiliated to Guangdong Medical University, Yangjiang, Guangdong, China; ^2^Key Laboratory of Respiratory Disease of Yangjiang, People’s Hospital of Yangjiang Affiliated to Guangdong Medical University, Yangjiang, Guangdong, China; ^3^Yangjiang Branch of Biochip Beijing National Engineering Research Center, People’s Hospital of Yangjiang Affiliated to Guangdong Medical University, Yangjiang, Guangdong, China; ^4^Department of Respiratory Diseases and Intensive Care Medicine, People’s Hospital of Yangjiang Affiliated to Guangdong Medical University, Yangjiang, Guangdong, China; ^5^Department of Oncology, People’s Hospital of Yangjiang Affiliated to Guangdong Medical University, Yangjiang, Guangdong, China

**Keywords:** COVID-19, Omicron, cancer, clinical characteristics, China

## Abstract

**Objective:**

To analyze the clinical characteristics and prognostic impacts of SARS-CoV-2 Omicron infection among cancer inpatients during the December 2022 – February 2023 surge, in order to provide scientific evidence for clinical treatment and prevention and control measures.

**Methods:**

A retrospective analysis was conducted on the clinical features, prognosis, and vaccination status of cancer in-patients infected with the Omicron variant during the COVID-19 pandemic of December 2022 – February 2023.

**Results:**

A total of 137 cancer inpatients were included in the study, with a median age of 61 years, and 75 patients (54.74%) were male. The main symptoms were cough (69 cases, 50.36%), expectoration (60 cases, 43.80%), and fever (53 cases, 39.69%). Chest CT examination revealed bilateral pneumonia in 47 cases (34.31%, 47/137) and pleural effusion in 24 cases (17.52%, 24/137). Among the cancer patients, 116 cases (84.67%, 116/137) had solid tumors, and 21 cases (15.33%, 21/137) had hematologic malignancies, with the main types being breast cancer (25 cases, 18.25%) and lung cancer (24 cases, 17.52%). Among the cancer patients, 46 cases (33.58%) were asymptomatic, 81 cases (59.12%) had mild disease, 10 cases (7.30%) had severe infection, and 8 cases (5.84%) died. A total of 91 patients (66.42%) had been vaccinated, with 58 patients (42.34%) receiving three doses. Multivariate analysis showed that cerebral infarction and hypoproteinemia were risk factors for death from COVID-19 infection.

**Conclusion:**

Cancer patients infected with SARS-CoV-2 Omicron typically exhibit mild disease manifestations, but some cancer patients infected with the Omicron variant might progress to severe illness, and even death, necessitating close monitoring and attention during the early stages of infection. Additionally, the presence of cerebral infarction and hypoproteinemia significantly increases the risk of death.

## 1 Introduction

Since the outbreak of the coronavirus (COVID-19) in 2019, the SARS-CoV-2 virus has been raging globally for nearly 3 years, exerting a significant impact on the global economy and bringing unprecedented disease burdens and public health pressures. As of 1 January 2023, the world has reported over 656 million confirmed cases and more than 6.6 million deaths (1). The virus spreads primarily through interpersonal transmission and new variants continue to emerge. The Omicron variant was first detected in South Africa on 9 November 2021, and subsequently rapidly disseminated globally (2). Due to its enhanced transmissibility and immune evasion capabilities (3–5), Omicron easily spreads through human mobility and contact.

Since December 2021, the Omicron strain has locally erupted in Tianjin, Shanghai, and other regions of China. Based on the several rounds of locally transmitted Omicron outbreaks that have occurred in China, the infection rate is high, with low hospitalization rates, lighter disease severity, and lower mortality rates (6–8). The COVID-19 pandemic continues to pose significant challenges worldwide, despite efforts to control its spread and impact. The global control of the COVID-19 pandemic is a multifaceted challenge that demands coordinated efforts, innovative solutions, and collective resilience. Addressing the ongoing challenges, such as vaccine distribution inequities, vaccine hesitancy, emergence of new variants, overwhelmed healthcare systems, and economic repercussions, requires a comprehensive approach that prioritizes equity, collaboration, and evidence-based interventions. By acknowledging these challenges and working together to overcome them, the world can advance toward a future free from the grip of the COVID-19 pandemic (9, 10).

In late 2022, the Chinese government adjusted its prevention and control policies, shifting from a “dynamic zero-COVID” strategy to normalization of epidemic prevention and control measures (11). This resulted in the termination of isolation measures for COVID-19 infected individuals, leading to the first large-scale COVID-19 outbreak in Yangjiang during December 2022 to February 2023. Cancer patients are vulnerable to SARS-CoV-2 infection due to their physical condition and compromised immune system, potentially leading to more severe disease manifestations and poorer prognoses (12, 13). Nevertheless, there is still a lack of extensive research on the effects of the Omicron variant on cancer patients in China (14, 15). Therefore, this study aims to analyze the infection characteristics, outcomes, and vaccination status of this special population during the COVID-19 Omicron pandemic, providing scientific evidence for clinical treatment and prevention and control strategies.

## 2 Materials and methods

### 2.1 Study population

A retrospective analysis was conducted on cancer patients infected with the Omicron variant of SARS-CoV-2 and hospitalized in a tertiary hospital in western Guangdong between 8 December 2022, and 14 February 2023. Adult patients (≥18 years old) diagnosed with cancer and hospitalized were included in the study, including solid tumors, *in situ* cancers, and hematologic malignancies. Patients with benign tumors or those who had undergone curative surgery without recurrence for 5 years were excluded from the analysis. All patients tested positive for SARS-CoV-2 nucleic acid through real-time reverse transcription polymerase chain reaction (RT-PCR) using nasopharyngeal swab samples. The diagnosis and clinical classification of COVID-19 followed the diagnostic criteria outlined in the “Diagnosis and Treatment Plan for Novel Coronavirus Pneumonia (10th trial edition),” categorizing patients into asymptomatic, mild, moderate, severe, and critical types (16). For a better understanding of clinical features, this study grouped mild and moderate cases as non-severe group, and severe and critical cases as severe group. This study was approved by the Research Ethics Committee of Yangjiang People’s Hospital (No. 20230003).

### 2.2 Study method

Patient medical records and vaccination information were collected, including age, sex, clinical characteristics, cancer types, laboratory test results (complete blood count, serum biochemistry, coagulation assays, and inflammatory markers), and pulmonary imaging findings (CT scans and X-rays). Clinical symptoms and laboratory indices were collected at the time of the first hospital admission test. If data were not available at admission, the most recent test results shortly after admission were used. Information on drug treatments received by hospitalized patients, including antiviral drugs, antibiotics, anticoagulants, and corticosteroids, was also gathered. When necessary, patients will receive oxygen therapy, including low-flow, medium-flow oxygen, high-flow oxygen, non-invasive ventilation, and invasive ventilation with endotracheal intubation. Two outcomes were evaluated: discharge and death. Death was defined as mortality during hospitalization and death within 1 week of discharge due to critical condition and abandonment of treatment.

### 2.3 Statistical analysis

Data analysis was performed using SPSS 22.0 software. Continuous variables and categorical variables were represented as median, count (*n*), and percentage (%), while numerical data were described using mean ± standard deviation (x̄ ± s). Group data were analyzed using independent sample *t*-tests. Multifactor analysis was employed to explore factors influencing mortality in COVID-19 infections, and *P* < 0.05 was considered statistically significant.

## 3 Results

### 3.1 Basic characteristics and clinical data of cancer patients

A total of 137 patients were included, with a median age of 61 years (range: 26–95 years), and 51.1% of the patients were over 60 years old, with 54.74% (74/137) being male. The age distribution was mainly concentrated in the middle-aged and elderly stages, with the most patients falling between the ages of 51–70, totaling 75 individuals, accounting for over half (54.8%). Younger patients (18–40 years) and elderly patients (over 80 years) were relatively fewer, accounting for 5.1% (7 cases) and 13.9% (19 cases), respectively. Major symptoms included cough (50.36%, 69/137), expectorations (43.80%, 60/137), and fever (38.69%, 53/137). Some patients also experienced fatigue (20.44%, 28/137), sore throat (16.79%, 23/137), loss of appetite (16.79%, 23/137), dizziness/headache (14.60%, 20/137), and shortness of breath (13.87%, 19/137). Among the 137 patients, 33.58% (46/137) were asymptomatic, 59.12% (81/137) had non-severe symptoms, and 7.30% (10/137) were classified as severe. A total of 54.75% (75/137) of the patients had one or more underlying medical conditions, with hypoalbuminemia (25.55%, 35/137), hypertension (21.90%, 30/137), and anemia (21.17%, 29/137) being the most common. Radiological findings mostly indicated bilateral pneumonia (34.31%, 47/137), with a few cases showing bilateral pleural effusion (13.14%, 18/137) and unilateral pleural effusion (4.38%, 6/137) (Table 1).

**TABLE 1:** Clinical characteristics and laboratory results of COVID-19 in cancer patients.

	Total	Asymptomatic group	Mild symptom group	Severe symptom group	*P*-value
Number of patients	137 (100.00%)	46 (33.58%)	81 (59.12%)	10 (7.30%)	
Age (median; min–max)	61 (26–95)	59 (26–92)	63 (26–89)	67 (28–95)	0.075
Age groups					0.634
≤50	24 (17.52%)	9 (19.57%)	14 (17.28%)	1 (10.00%)	
51–60	43 (31.39%)	15 (32.61%)	24 (29.63%)	4 (40.00%)	
61–70	32 (23.36%)	12 (26.09%)	20 (24.69%)	0	
71–80	19 (13.87%)	5 (10.87%)	12 (14.81%)	2 (20.00%)	
>80	19 (13.87%)	5 (10.87%)	11 (13.58%)	3 (30.00%)	
Sex					0.01
Male	75 (54.74%)	25 (54.35%)	40 (49.38%)	10 (100.00%)	
Female	62 (45.26%)	21 (45.65%)	41 (50.62%)	0	
**Clinical symptoms**					
Fever	53 (38.69%)	0	46 (56.79%)	7 (70.00%)	<0.01
Peak temperature (°C)	41	–	41	39.6	–
Cough	69 (50.36%)	1 (2.17%)	60 (74.07%)	8 (80.00%)	<0.01
Expectoration	60 (43.80%)	1 (2.17%)	50 (61.73%)	9 (90.00%)	<0.01
Rhinorrhea	5 (3.65%)	1 (2.17%)	4 (4.94%)	0	0.593
Nasal congestion	3 (2.19%)	1 (2.17%)	2 (2.47%)	0	0.881
Sore throat	23 (16.79%)	1 (2.17%)	22 (27.16%)	0	<0.01
Hoarseness	1 (0.73%)	0	1 (1.23%)	0	0.706
Loss of appetite	23 (16.79%)	2 (4.35%)	16 (19.75%)	5 (50.00%)	0.001
Diarrhea	1 (0.73%)	0	1 (1.23%)	0	0.706
General fatigue	28 (20.44%)	1 (2.17%)	23 (28.40%)	4 (40.00%)	0.001
Dizziness/headache	20 (14.60%)	0	18 (22.22%)	2 (20.00%)	0.003
Muscle soreness	4 (2.92%)	0	4 (4.94%)	0	0.241
Abdominal pain	7 (5.11%)	1 (2.17%)	6 (7.41%)	0	0.327
Nausea	7 (5.11%)	0	7 (8.64%)	0	0.078
Vomiting	5 (3.65%)	0	5 (6.17%)	0	0.166
Chest tightness	11 (8.03%)	1 (2.17%)	7 (8.64%)	3 (30.00%)	0.013
Shortness of breath	19 (13.87%)	0	13 (16.05%)	6 (60.00%)	<0.01
**Underlying diseases**					
Hypertension	30 (21.90%)	5 (10.87%)	21 (25.93%)	4 (40.00%)	0.051
Diabetes	11 (8.03%)	2 (4.35%)	6 (7.41%)	3 (30.00%)	0.024
Cerebral infarction	8 (5.84%)	1 (2.17%)	5 (6.17%)	2 (20.00%)	0.091
Coronary heart disease	10 (7.30%)	1 (2.17%)	8 (9.88%)	1 (10.00%)	0.261
COPD	4 (2.92%)	0	2 (2.47%)	2 (20.00%)	0.003
Chronic gastritis/gastric ulcer	7 (5.11%)	1 (2.17%)	6 (7.41%)	0	0.327
Chronic kidney disease	8 (5.84%)	1 (2.17%)	6 (7.41%)	1 (10.00%)	0.406
Chronic liver disease	22 (16.06%)	11 (23.91%)	8 (9.88%)	3 (30.00%)	0.054
Hypoproteinemia	35 (25.55%)	9 (19.57%)	19 (23.46%)	7 (70.00%)	0.003
Anemia	29 (21.17%)	7 (15.22%)	19 (23.46%)	3 (30.00%)	0.428
Number of underlying diseases					0.004
1	25 (18.25%)	9 (19.57%)	15 (18.52%)	1 (10.00%)	
2	25 (18.25%)	7 (15.22%)	15 (18.52%)	3 (30.00%)	
3 and above	25 (18.25%)	4 (8.70%)	15 (18.52%)	6 (60.00%)	
Duration of hospitalization (median days)	10	7	11	25	<0.01
Death	8 (5.84%)	0	1 (1.23%)	7 (70.00%)	
Radiological findings (*N* = 53)					0.155
Unilateral pneumonia	4 (2.92%)	0	4 (4.94%)	0	
Bilateral pneumonia	47 (34.31%)	1 (2.17%)	37 (45.68%)	9 (90.00%)	
Unilateral pleural effusion	6 (4.38%)	1 (2.17%)	4 (4.94%)	1 (10.00%)	
Bilateral pleural effusion	18 (13.14%)	0	14 (17.28%)	4 (40.00%)	
Electrocardiogram results (*N* = 69)					0.038
Normal	13 (9.49%)	1 (2.17%)	11 (13.58%)	1 (10.00%)	
Abnormal	56 (40.88%)	3 (6.52%)	47 (58.02%)	6 (60.00%)	
Respiratory support (*N* = 59)					<0.01
Oxygen supply	59 (43.07%)	1 (2.17%)	49 (60.49%)	9 (90.00%)	
Low-flow oxygen	8 (5.84%)	0	8 (9.88%)	0	
Medium-flow oxygen	39 (28.47%)	1 (2.17%)	38 (46.91%)	0	
High-flow oxygen	3 (2.19%)	0	2 (2.47%)	1 (10.00%)	
Noninvasive ventilation	2 (1.46%)	0	0	2 (20.00%)	
Invasive mechanical ventilation	7 (5.11%)	0	1 (1.23%)	6 (60.00%)	
**Treatments**					
Antibiotic therapy	59 (43.07%)	1 (2.17%)	50 (61.73%)	8 (80.00%)	<0.01
Antiviral therapy	9 (6.57%)	0	8 (9.88%)	1 (10.00%)	0.088
Anticoagulant therapy	9 (6.57%)	0	7 (8.64%)	2 (20.00%)	0.034
Hormone therapy	34 (24.82%)	1 (2.17%)	26 (32.10%)	7 (70.00%)	<0.01

Data presented as median or *n* (%), *P* < 0.05 indicates statistically significant difference. COPD, chronic obstructive pulmonary disease.

The average length of hospital stay was 10 days, with a longer duration noted in patients experiencing more severe symptoms. Out of eight patients (5.84%) who passed away, 70% (7/10) had severe cases of COVID-19. Five deaths resulted from respiratory failure and severe pneumonia, while three fatalities were not directly linked to COVID-19.

### 3.2 Types of cancer

A total of 84.67% (116/137) of the patients had solid tumors, while 15.33% (21/137) had hematologic malignancies. The most common cancer types were breast cancer (18.25%, 25/137), lung cancer (17.52%, 24/137), colorectal cancer (10.22%, 14/137), nasopharyngeal cancer (8.03%, 11/137), and liver cancer (8.03%, 11/137). Among solid tumor patients, 60.34% (70/116) were in advanced stages of cancer, and 38.79% (45/116) had already metastasized. There was no statistically significant association between cancer type and COVID-19 severity classification (Table 2).

**TABLE 2:** Cancer types.

Cancer type	Total	Asymptomatic group	Mild group	Severe group	*P*-value
Total	137 (100.00%)	46 (33.58%)	81 (59.12%)	10 (7.30%)	0.12
Hematological malignancies	21 (15.33%)	2 (4.35%)	18 (22.22%)	1 (10.00%)	
Multiple myeloma	7 (5.11%)	1 (2.17%)	6 (7.41%)	0	
Acute myeloid leukemia	5 (3.65%)	0	5 (6.17%)	0	
Lymphoma	5 (3.65%)	0	4 (4.94%)	1 (10.00%)	
Chronic lymphocytic leukemia	2 (1.46%)	0	2 (2.47%)	0	
Myelodysplastic syndrome	1 (0.73%)	0	1 (1.23%)	0	
Primary myelofibrosis	1 (0.73%)	1 (2.17%)	0	0	
Solid tumors	116 (84.67%)	44 (95.65%)	63 (77.78%)	9 (90.00%)	
Breast cancer	25 (18.25%)	9 (19.57%)	16 (19.75%)	0	
Lung cancer	24 (17.52%)	5 (10.87%)	18 (22.22%)	1 (10.00%)	
Colorectal cancer	14 (10.22%)	9 (19.57%)	4(4.94%)	1 (10.00%)	
Nasopharyngeal cancer	11 (8.03%)	4 (8.70%)	5 (6.17%)	2 (20.00%)	
Liver cancer	11 (8.03%)	7 (15.22%)	3 (3.70%)	1 (10.00%)	
Prostate cancer	10 (7.30%)	3 (6.52%)	5 (6.17%)	2 (20.00%)	
Cervical cancer	5 (3.65%)	2 (4.35%)	3 (3.70%)	0	
Gastric cancer	4 (2.92%)	2 (4.35%)	2 (2.47%)	0	
Esophageal cancer	3 (2.19%)	0	3 (3.70%)	0	
Bladder cancer	2 (1.46%)	0	1 (1.23%)	1 (10.00%)	
Other cancers*	7 (5.11%)	3 (6.52%)	3 (3.70%)	1 (10.00%)	
**Solid tumor stage**					
Early	34 (29.31%)	11 (25.00%)	21 (33.33%)	2 (22.22%)	0.636
Mid-stage	12 (10.34%)	5 (11.36%)	7 (11.11%)	0	
Advanced	70 (60.34%)	28 (63.64%)	35 (55.56%)	7 (77.78%)	
Metastatic	45 (38.79%)	16 (36.36%)	24 (38.10%)	5 (55.56%)	0.552

*Other cancer types include one case each of malignant peritoneal mesothelioma, malignant tumor around ampulla of Vater, thyroid cancer, ovarian cancer, brain stem glioma, renal cancer, and pancreatic cancer. Data presented as *n* (%), *P* < 0.05 indicates a statistically significant difference.

### 3.3 Vaccine administration status

Over 66.42% (91/137) of cancer patients have received the COVID-19 vaccine, with 42.34% (58/137) having received all three doses. The earliest vaccination date was on 26 March 2021, and the latest was on 23 December 2022, with vaccination ages ranging from 25 to 80 years old. A total of 54.95% (50/91) of the vaccines administered were produced by Sinovac Biotech Ltd. There was no significant association found between vaccine administration and the severity of COVID-19 cases. Details of vaccine administration based on COVID-19 severity classification can be found in Table 3.

**TABLE 3:** COVID-19 vaccination status.

Category	Total	Asymptomatic group	Mild group	Severe group	*P*-value
Total	137 (100.00%)	46 (33.58%)	81 (59.12%)	10 (7.30%)	0.27
Unvaccinated	46 (33.58%)	14 (30.43%)	26 (44.44%)	6 (60.00%)	0.18
Vaccinated	91 (66.42%)	32 (69.57%)	55 (67.90%)	4 (40.00%)	0.18
1 dose	9 (6.57%)	1 (2.17%)	7 (8.64%)	1 (10.00%)	0.32
2 doses	24 (17.52%)	7 (15.22%)	15 (18.52%)	2 (20.00%)	0.87
3 doses	58 (42.34%)	24 (52.17%)	33 (40.74%)	1 (10.00%)	0.05
Last dose inoculation before infection					0.86
<6 months	7 (5.11%)	1 (2.17%)	6 (7.41%)	0	0.35
6 months–1 year	50 (36.5%)	20 (43.48%)	28 (34.57%)	2 (20.00%)	0.56
>1 year	34 (24.82%)	11 (23.91%)	21 (25.93%)	2 (20.00%)	0.81

Data presented as *n* (%). *P* < 0.05 indicates a statistically significant difference.

### 3.4 Comparison of laboratory results in solid tumor patients

Due to significant variations in blood results among patients with hematologic malignancies, statistical analysis was not deemed meaningful. Therefore, solid tumor patients were divided into two groups based on COVID-19 severity classification: non-severe group including asymptomatic, mild, and moderate cases, and severe group comprising severe and critical cases. In cases of multiple laboratory results, the data from the first hospital admission test were documented. Statistically significant differences were observed in various laboratory parameters between the severe and non-severe groups of COVID-19 patients, including neutrophils (Neu), NLR (neutrophils to lymphocytes ratio), aspartate aminotransferase (AST), urea, creatinine (CR), hydroxybutyrate dehydrogenase (HBDH), lactate dehydrogenase (LDH), C-reactive protein (CRP), interleukin-8 (IL-8), lymphocytes (Lym), total protein (TP), and albumin (ALB). Results of the mentioned parameters were generally higher in the severe group than in the non-severe group of COVID-19 patients, particularly in WBC count, Neu count, and CRP, which are associated with infection and inflammation. On the other hand, levels of RBC and Lym were lower in the severe group compared to the non-severe group, potentially indicating compromised immune function (Table 4).

**TABLE 4:** Laboratory indices of severe and non-severe solid tumor patients upon hospital admission.

Clinical features	All (*n*)	Non-severe (*n*)	Severe (*n*)	*P*-value
RBC (×10^12^/L)	3.83 ± 0.88 (114)	3.84 ± 0.88 (105)	3.63 ± 0.87 (9)	0.59
HGB (g/L)	109.17 ± 22.92 (114)	109.14 ± 22.99 (105)	109.44 ± 23.44 (9)	0.92
PLT (×10^9^/L)	214.17 ± 104.12 (114)	214.67 ± 106.8 (105)	208.33 ± 69.29 (9)	0.85
WBC (×10^9^/L)	7.08 ± 4.26 (114)	6.8 ± 3.87 (105)	10.41 ± 6.96 (9)	0.11
Neu (×10^9^/L)	5.41 ± 4.01 (114)	5.09 ± 3.62 (105)	9.17 ± 6.28 (9)	0.03
Lym (×10^9^/L)	1.02 ± 0.58 (114)	1.05 ± 0.58 (105)	0.65 ± 0.41 (9)	0.03
Mon (×10^9^/L)	0.6 ± 0.35 (114)	0.6 ± 0.34 (105)	0.53 ± 0.38 (9)	0.58
NLR	7.54 ± 8.93 (114)	6.87 ± 8.79 (105)	15.29 ± 7.01 (9)	<0.01
MLR	0.72 ± 0.55 (114)	0.71 ± 0.55 (105)	0.87 ± 0.63 (9)	0.71
PLR	278.79 ± 240.25 (114)	265.41 ± 228.73 (105)	434.79 ± 324.77 (9)	0.04
D-dimer (mg/L)	2.67 ± 4.46 (95)	2.16 ± 3.76 (86)	7.54 ± 7.32 (9)	0.03
ALT (U/L)	34.46 ± 32.37 (113)	33.7 ± 31.12 (104)	43.27 ± 45.89 (9)	0.8
AST (U/L)	46.87 ± 57.32 (113)	43.75 ± 53.21 (104)	82.51 ± 89.02 (9)	0.02
ALP (U/L)	107.74 ± 99.35 (113)	105.66 ± 98.34 (104)	131.78 ± 113.88 (9)	0.48
GGT (U/L)	62.11 ± 95.93 (113)	56.32 ± 87.62 (104)	129.09 ± 156.53 (9)	0.51
TBIL (μmol/L)	19.66 ± 65.73 (113)	14.26 ± 23.28 (104)	82.08 ± 220.8 (9)	0.59
DBIL (μmol/L)	11.26 ± 50.4 (113)	6.98 ± 16.27 (104)	60.77 ± 170.76 (9)	0.29
IBIL (μmol/L)	8.37 ± 15.71 (113)	7.25 ± 7.6 (104)	21.31 ± 50.06 (9)	0.68
TP (g/L)	66.07 ± 8.99 (113)	66.98 ± 7.99 (104)	55.53 ± 13.18 (9)	0.01
ALB (g/L)	36.26 ± 10.78 (113)	36.83 ± 10.94 (104)	29.64 ± 5.76 (9)	0.01
GLOB (g/L)	30.63 ± 5.82 (113)	30.88 ± 5.78 (104)	27.77 ± 5.88 (9)	0.17
A/G	1.55 ± 3.85 (113)	1.58 ± 4.01 (104)	1.1 ± 0.25 (9)	0.2
Urea (mmol/L)	6.34 ± 5.17 (114)	6.08 ± 5.13 (105)	9.28 ± 5.03 (9)	0.01
Cr (mg/L)	86.36 ± 92.99 (114)	85.54 ± 96.36 (105)	95.89 ± 36.19 (9)	0.01
Urea/Cr	0.09 ± 0.1 (114)	0.08 ± 0.1 (105)	0.1 ± 0.05 (9)	0.1
CK-MB (U/L)	24.95 ± 26.66 (104)	24.4 ± 26.48 (96)	31.54 ± 29.91 (8)	0.46
CK (U/L)	203.07 ± 436.59 (103)	173.78 ± 297.41 (95)	550.88 ± 1,199.92 (8)	0.11
HBDH (U/L)	212.45 ± 113.66 (104)	205.88 ± 111.18 (96)	291.25 ± 121.01 (8)	0.01
LDH (U/L)	329.15 ± 574.18 (107)	267.97 ± 149.24 (99)	1,086.25 ± 1,993.11 (8)	<0.01
CRP (mg/L)	39.33 ± 51.56 (103)	34.39 ± 46.8 (95)	97.93 ± 71.24 (8)	<0.01
GLU (mmol/L)	6.53 ± 2.88 (108)	6.35 ± 2.47 (99)	8.56 ± 5.61 (9)	0.58
IL-10 (pg/ml)	14.23 ± 22.84 (29)	9.72 ± 15.04 (21)	29.99 ± 37.74 (8)	0.06
IL-6 (pg/ml)	193.28 ± 337.86 (29)	139.77 ± 241.82 (21)	380.57 ± 552.78 (8)	0.07
IL-8 (pg/ml)	76.07 ± 94.91 (29)	61.02 ± 90.68 (21)	128.76 ± 98.32 (8)	0.04
BNP (pg/ml)	3,134.92 ± 6,848.92 (35)	3,426.39 ± 7,590.86 (28)	1,969.06 ± 2,128.99 (7)	0.59
CTnI (ng/ml)	22.41 ± 34.95 (31)	18.95 ± 31.55 (25)	36.83 ± 47.35 (6)	0.25

Data presented as mean ± SD and n represents the number of patients with available data. *P* < 0.05 is considered statistically significant. RBC, red blood cell; HGB, hemoglobin; PLT, platelet; Neu, neutrophil count; Lym, lymphocyte count; Mon, monocyte count; NLR, neutrophils to lymphocytes ratio; MLR: monocyte to lymphocyte ratio; PLR, platelet to lymphocytes ratio; D-dimer, D-dimer test; ALT, alanine aminotransferase; AST, aspartate aminotransferase; ALP, alkaline phosphatase; GGT, gamma-glutamyl transferase; TBIL, total bilirubin; DBIL, direct bilirubin; IBIL, indirect bilirubin; TP, total protein; ALB, albumin; GLOB, globulin; A/G, albumin-globulin ratio; Urea, urea nitrogen; Cr, creatinine; urea/Cr, urea-to-creatinine ratio; CK-MB, creatine kinase MB; CK, creatine kinase; HBDH, hydroxybutyrate dehydrogenase; LDH, lactate dehydrogenase; CRP, C-reactive protein; GLU, glucose; IL, interleukin; BNP, brain natriuretic peptide; CTnI, cardiac troponin I.

### 3.5 High-risk factors for COVID-19 mortality

There were a total of 8 cancer patients who died, spanning a wide age range from 28 to 95 years, all of whom were male. The shortest hospitalization period among these patients was 1 day, while the longest was 58 days. The types of cancer varied and included prostate cancer, bladder cancer, lymphoma, nasopharyngeal cancer, brainstem glioma, liver cancer, and lung cancer, predominantly in advanced stages. Based on COVID-19 severity classification, seven of the deceased cases were critical, while one was mild, and a total of five patients required endotracheal intubation and mechanical ventilation assistance for breathing. All patients had other comorbidities and health issues, with COVID-19 infection being a significant contributing factor to their deaths (Supplementary Table 1).

In the linear regression model exploring risk factors for COVID-19 mortality, individuals with cerebral infarction and hypoalbuminemia have a significantly elevated risk of death (Table 5).

**TABLE 5:** Analysis of risk factors for COVID-19 mortality.

Variable	Coefficient estimate	SE	t-Value	*P*-value
(Intercept)	−6.313	3.925	−1.609	0.108
Age	0.006	0.034	0.169	0.865
Sex (female)	−21.51	2,613	−0.008	0.993
Solid tumor/blood disorder	−1.45	2.044	−0.709	0.478
Diabetes	4.281	2.31	1.853	0.06
Cerebral infarction	4.31	2.102	2.05	0.04*
Coronary heart disease	−0.207	1.806	−0.114	0.909
COPD	1.631	2.103	0.776	0.438
Chronic gastric disease	−15.08	8,403	−0.01	0.999
Chronic kidney disease	3.593	2.236	1.607	0.108
Chronic liver disease	1.1	1.46	0.754	0.451
Hypoproteinemia	3.357	1.481	2.268	0.02*
Smoking	1.944	1.985	0.979	0.3276
Vaccination	2.454	2.505	0.98	0.327

*P* < 0.05 is considered statistically significant. COPD, chronic obstructive pulmonary disease.

*Indicates statistical significance.

## 4 Discussion

Since the outbreak of the COVID-19 pandemic at the end of 2019, the China government has implemented a dynamic Zero COVID-19 strategy, this policy entails swiftly dealing with each discovered local confirmed case, extinguishing the infection promptly to eradicate the transmission chain entirely—a strategy commonly known as achieving zero cases (11). Since December 2019, a series of domestic outbreaks has occurred in the country. Whenever confirmed cases emerge, strict control measures are implemented at their activity venues and residences. Multiple rounds of nucleic acid testing are conducted, and only locations free from epidemic risks are unsealed, aligning with the overarching strategy for COVID-19 epidemic prevention and control. This comprehensive approach encompasses three key elements: timely and proactive identification of infection sources, swift implementation of public health and social intervention measures to trace and manage close contacts, and effective patient treatment. While China’s “Dynamic Zero” epidemic prevention policy has yielded significant results in epidemic control, the localized closed control measures have led to factory shutdowns and logistical disruptions, exerting a noticeable adverse impact on economic operations (17). In order to better cope with virus mutations and changes in the epidemic situation, by the end of 2022, significant adjustments were made to epidemic prevention and control policies in China, including reducing lockdown durations, optimizing isolation methods, relaxing entry policies, and enhancing vaccine coverage (18). The number of domestic SARS-CoV-2 infections has significantly increased, marking the country’s first nationwide COVID-19 pandemic, as well as the first large-scale local outbreak in the Yangjiang region. Monitoring data from the Chinese Center for Disease Control and Prevention shows that since 9 December 2022, a nationwide pandemic of COVID-19 infections has emerged, with the number of positive cases peaking on December 22 (6.94 million) before gradually declining. During this pandemic, the predominant strains across the country are the BA.5.2 and BF.7 variants of the Omicron mutant (19).

Since the discovery of the COVID-19 Omicron variant in South Africa in November 2021, it has rapidly spread globally due to its high transmissibility and immune evasion capabilities (2–5). In early 2022, the Omicron strain began to emerge in localized areas in China, with studies in Macau and Shanghai indicating that individuals infected with the Omicron variant may experience relatively mild symptoms and could potentially present with more asymptomatic infections (8, 19).

According to previous research reports, cancer patients are considered a high-risk group for COVID-19, with a higher risk of infection, increased susceptibility to severe illness, and higher mortality rates (20, 21). Various risk factors are associated with the severity of COVID-19 in cancer patients, including age, underlying health conditions, and cancer types (22, 23). In this study, the male-to-female ratio of cancer patients was 1.2:1, with 82.48% (113/137) of patients over the age of 50, 33.58% (46/137) being asymptomatic carriers, 59.12% (81/137) having mild symptoms, and 7.30% (10/137) in critical condition. Out of the total patients, eight died, with seven being severe/critical COVID-19 cases, of which five deaths were due to respiratory failure, severe pneumonia, and thee deaths were not directly attributed to COVID-19. Our study found a mortality rate of 5.84% (8/137) in cancer patients with COVID-19 infection, while the mortality rate in the severe/critical COVID-19 group was 70% (7/10). Additionally, this study found that cancer patients with conditions such as stroke and hypoalbuminemia may face significantly higher mortality risks. Consistent with the findings of Lee, Sun, and other scholars, for cancer patients, especially those with multiple comorbidities, Omicron infection may still lead to serious outcomes (24, 25). Previous research has indicated that patients with hematologic cancers may be at a higher risk of severe COVID-19 due to immune system alterations in blood cell function compared to non-hematologic cancer patients (26). However, due to the small sample size in this study, there was insufficient data to explore the relationship between the type of cancer and the severity of COVID-19.

In our study, the clinical manifestations of cancer patients combined with COVID-19 infection were similar to the general population, primarily presenting symptoms such as cough, sputum production, and fever (6, 7, 12). However, significant differences were observed in laboratory tests between cancer patients with severe COVID-19 infection and non-severe cases. Specifically, levels of indicators such as Neu, AST, Urea, CR, HBDH, LDH, CRP, and IL-8 were elevated in severe patients, while levels of indicators like LY, TP, and ALB were decreased. These differences may be related to the severity of the patient’s illness and any comorbidities (27). For cancer patients with concurrent COVID-19 infection, close monitoring of their condition and implementation of effective treatment measures are crucial to reduce the incidence and mortality rates of severe cases. Additionally, preventive measures are also essential to decrease the risk of infection.

Vaccination in China primarily consists of inactivated vaccines, adenovirus vector vaccines, and recombinant subunit protein vaccines, with a higher proportion of individuals receiving inactivated vaccines. Over 90% of Chinese citizens have completed the two doses of inactivated COVID-19 vaccines produced by Sinovac Biotech and China National Pharmaceutical Group (28), with no significant differences observed between different vaccine types (29, 30). In this study, 66.42% (91/137) of cancer patients had been vaccinated against COVID-19, although the protective effect of the vaccine was not significant, likely due to the small sample size. While vaccination may not provide optimal protection against the Omicron variant, it can reduce the likelihood of severe illness and death compared to unvaccinated individuals (31, 32). From the perspective of Chinese society, the COVID-19 vaccination program is still considered effective and cost-effective (28, 33), therefore, routine and booster doses of inactivated virus vaccines are necessary (34–36).

As the first comprehensive reopening after the COVID-19 pandemic in China, the mortality rate among cancer patients, a special high-risk group, has significantly decreased compared to previous reports, but still remains higher than the general population (37–39). Due to the shortage of hospital beds during the initial COVID-19 outbreak in the region, hospitals admitted only more severe cases, while many asymptomatic or mildly symptomatic cancer patients chose to self-isolate at home, waiting for improvement before receiving relevant treatment. In reality, the mortality rate among cancer patients with concurrent COVID-19 infection is lower (40). According to the announcement by the World Health Organization on 5 May 2023, the COVID-19 pandemic no longer constitutes a “Public Health Emergency of International Concern.” However, due to the continued variation of the novel coronavirus and the risk of reinfection (5, 41), effective epidemic prevention and control measures still need to be implemented, along with active virus research and treatment efforts.

In our study, we acknowledge certain limitations. Primarily, our sample was restricted to cancer patients from a single hospital in the Yangjiang region, thus limiting the generalizability of our findings regarding the prevalence of COVID-19 infection among cancer patients. Secondly, the lack of data from some laboratory tests and clinical records may lead to biased data interpretation. Therefore, for a more scientifically sound analysis, it is important to include a larger sample size and conduct longer follow-ups to comprehensively understand the impact of SARS-CoV-2 on cancer patients’ outcomes.

## Data availability statement

The original contributions presented in this study are included in the article/Supplementary material, further inquiries can be directed to the corresponding author.

## Ethics statement

The studies involving humans were approved by the Research Ethics Committee of Yangjiang People’s Hospital (No. 20230003). The studies were conducted in accordance with the local legislation and institutional requirements. The ethics committee/institutional review board waived the requirement of written informed consent for participation from the participants or the participants’ legal guardians/next of kin because this study was retrospective, and there is no identifiable data in this article.

## Author contributions

L-LL: Investigation, Methodology, Validation, Writing – original draft. Y-WL: Software, Writing – original draft. X-HY: Data curation, Methodology, Validation, Writing – original draft. LR: Validation, Writing – original draft. B-GC: Investigation, Writing – original draft. GC: Validation, Writing – review & editing. G-KZ: Investigation, Writing – review & editing. L-YY: Conceptualization, Funding acquisition, Resources, Supervision, Writing – original draft, Writing – review & editing.
